# Supplementary material for: The Peak Plasma Concentration (Cmax)/Minimum Inhibitory Concentration (MIC) of bedaquiline and levofloxacin with special attention to the sputum conversion in the treatment of multidrug-resistant tuberculosis in Indonesia
Source: PLoS One. 2025 Dec 31;20(12):e0336210. doi: 10.1371/journal.pone.0336210 (PMC12755803; doi:10.1371/journal.pone.0336210)
Supplement: S1 File — (DOCX) [file pone.0336210.s001.docx]

Sample size

The planned total enrollment was 57 patients, based on calculations that the largest number of levofloxacin Cmax/MIC variable. This sample size yields 80% power to detect a relationship between levofloxacin Cmax/MIC and the proportion of patients who achieve sputum conversion. The ratio used was 1:2, estimating the difference in proportion between the two groups. These sample size assumptions were based on previously published studies that showed the difference of means between groups was small (for the Levofloxacin Cmax/MIC variable), and the difference of means between groups was large for the bedaquiline Cmax/MIC variable [1,2]. By using MedCalc software, the results are shown in **Fig 1** and **Fig 2**.


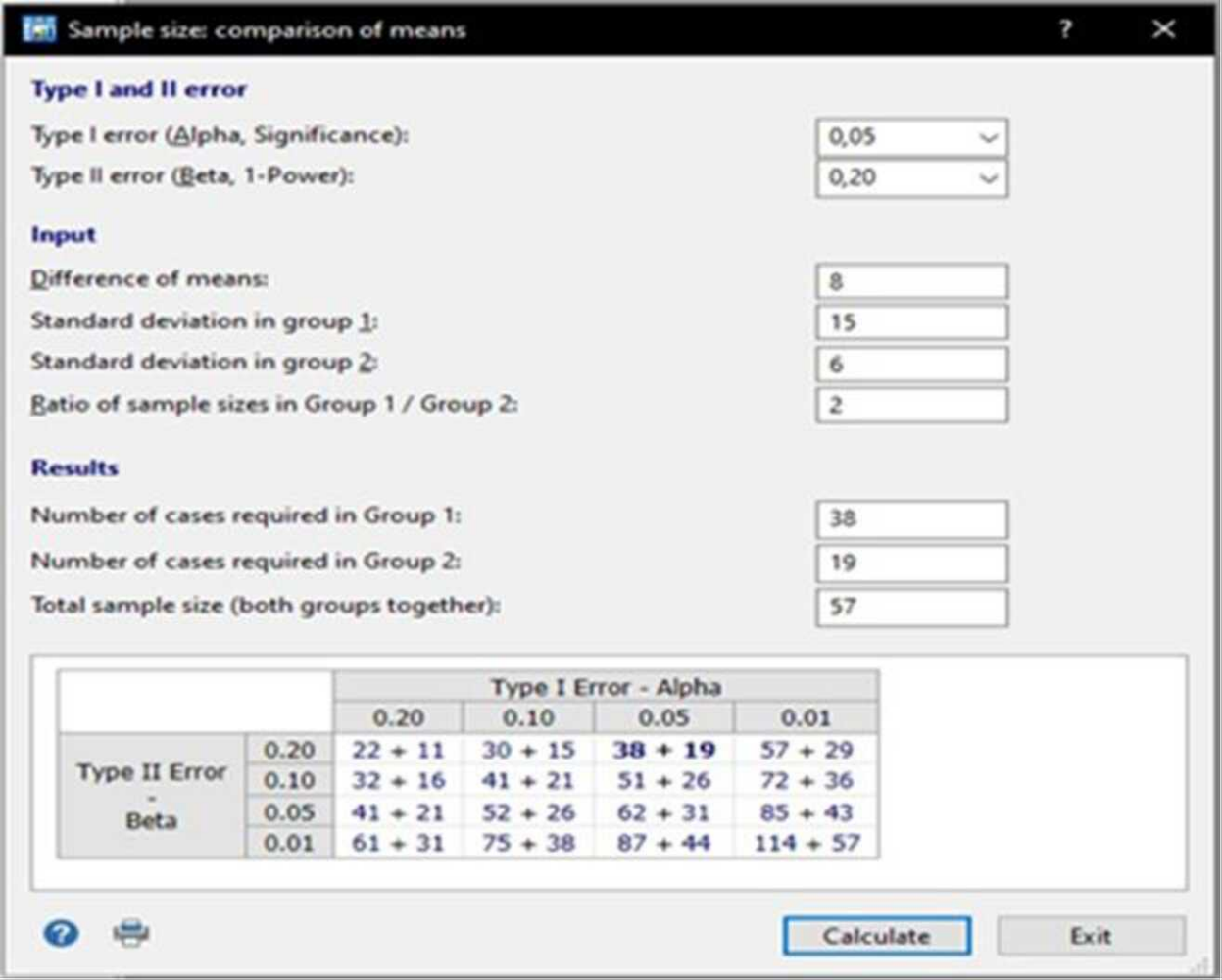


**Fig 1**. Sample Size Calculation Screenshot for Levofloxacin Cmax/MIC Ratio with Sputum Conversion Using Software MedCalc Ltd [3].


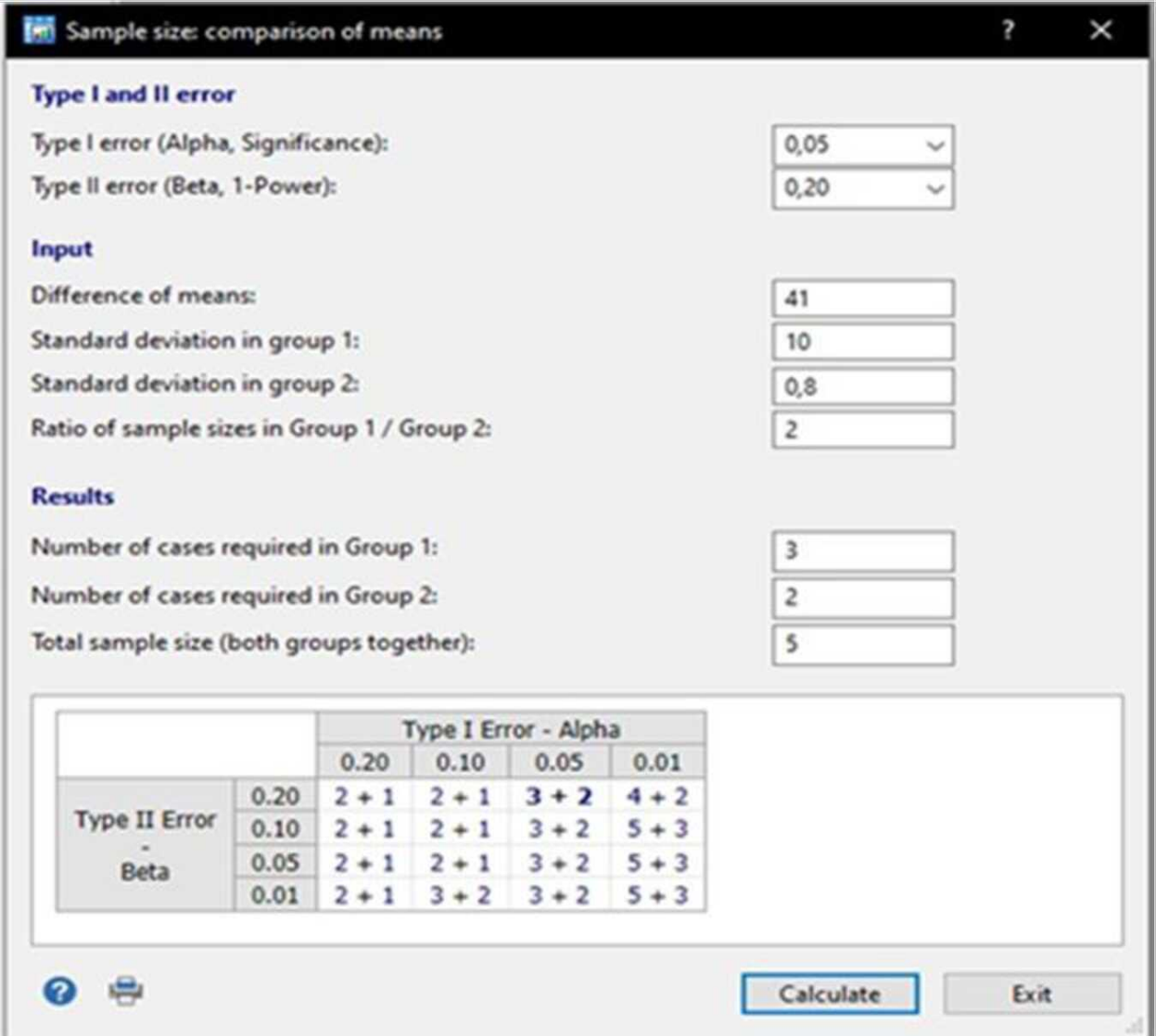


**Fig 2**. Sample Size Calculation Screenshot for Bedaquiline Cmax/MIC Ratio with Sputum Conversion Using Software MedCalc Ltd [3].

References:

1. Shao G, Bao Z, Davies Forsman L PJ, Werngren J, Niward K et al. Population pharmacokinetics and model-based dosing evaluation of bedaquiline in multidrug-resistant tuberculosis patients. Front Pharmacol. 2023;(14):1022090.

2. Mpagama SG, Ndusilo N, Stroup S, Kumburu H, Peloquin CA, Gratz J, et al. Plasma drug activity in patients on treatment for multidrug-resistant tuberculosis. Antimicrob Agents Chemother. 2014;58(2):782–8.

3. Machin D, Campbell MJ, Tan SB TS. Sample size calculation: Comparison of two means. In: Sample size tables for clinical studies. 3rd ed. Belgium: Chichester: Wiley-Blackwell; 2009.
