# Supplementary material for: The Peak Plasma Concentration (Cmax)/Minimum Inhibitory Concentration (MIC) of bedaquiline and levofloxacin with special attention to the sputum conversion in the treatment of multidrug-resistant tuberculosis in Indonesia
Source: PLoS One. 2025 Dec 31;20(12):e0336210. doi: 10.1371/journal.pone.0336210 (PMC12755803; doi:10.1371/journal.pone.0336210)
Supplement: S2 Table — (DOCX) [file pone.0336210.s002.docx]

**S2 Table. Demographic data and clinical characteristics of 58 MDR-TB patients at the start of treatment (baseline) by whether bedaquiline or levofloxacin Cmax is missing or not**

| **Characteristics** | **Levofloxacin and bedaquiline Cmax not missing** | **Missing levofloxacin or bedaquiline Cmax** | **p value** |
| --- | --- | --- | --- |
|  | **(n = 24)** | **(n = 34)** |  |
| Age (years), mean (SD) | 39 (13) | 38 (11) | 0.363^t^ |
| Sex, n (%) |  |  |  |
| Male | 17 (70.8) | 20 (58.9) | 0.559^f^ |
| Female | 7 (29.2) | 14 (41.2) |  |
| History of tobacco use, n (%) | 12 (50.0) | 20 (58.8) | 0.345^c^ |
| History of alcohol use, n (%) | 5 (20.8) | 6 (17.6) | 0.509^f^ |
| Previous TB treatment, n (%) | 14 (58.3) | 26 (76.5) | 0.119^c^ |
| Comorbidity (well-controlled diabetes) | 2 (8.3) | 0 | 0.167^f^ |
| Categorical BMI, n (%) |  |  |  |
| Normal weight (18.5 to 24.9 kg m^2^) | 11 (45.8) | 8 (23.5) | 0.067^c^ |
| Underweight (< 18.5 kg m^2^) | 31 (54.2) | 26 (76.5) |  |
| Lung lesion, n (%) |  |  |  |
| Moderate advanced | 2 (08.3) | 4 (11.8) | 0.514^f^ |
| Far advanced | 22 (91.7) | 30 (88.2) |  |
| Laboratory results |  |  |  |
| Hemoglobin (g/dL), mean (SD) | 12.2 (1.9) | 11.6 (1.7) | 0.226^t^ |
| Hematocrit (10^6/µL), mean (SD) | 38.7 (27–47) | 36.18 (5.48) | 0.347^t^ |
| Eritrosit (10^6/µL), mean (SD) | 4.8 (0.5) | 4.6 (0.5) | 0.287^t^ |
| Leukosit (µL), mean (SD) | 8900 (4072) | 10.647 (3703) | 0.095^t^ |
| Trombosit (10^3/µL), mean (SD) | 374 (138.7) | 385 (114.9) | 0.747^t^ |
| MCV (fL), mean (SD) | 81.1 (7.1) | 81.5 (5.5) | 0.526^t^ |
| MCH (pg), mean (SD) | 26 (3.3) | 26 (2.3) | 0.081^t^ |
| MCHC (g/dL), median (min-max) | 32.3 (2.1) | 32 (1.4) | 0.220^t^ |
| Neutrofil Limfosit Ratio, median (min-max) | 3.7 (1.4–9.4) | 4.4 (1.6–22.7) | 0.112^m^ |
| AST (IU/L), median (min-max) | 16.0 (10–45) | 21.5 (4–70) | 0.831^m^ |
| ALT (IU/L), median (min-max) | 13.5 (7–40) | 17.0 (5–66) | 0.593^m^ |
| Creatinine (mg/dL), mean (SD) | 0.9 (0.2) | 0.8 (0.2) | 0.147^t^ |
| Albumin (g/L), mean (SD) | 3.4 (0.6) | 3.3 (0.5) | 0.284^m^ |
| Other drug use, n (%) |  |  |  |
| Analgesics | 7 (29.2) | 10 (58.6) | 0.610^c^ |
| Proton Pump Inhibitor (PPI) | 9 (37.5) | 11 (32.4) | 0.448^c^ |
| Antiemetic | 1 (4.2) | 4 (11.8) | 0.304^f^ |
| Antifungal | 2 (8.3) | 1 (2.9) | 0.370^f^ |
| Antiulcerant | 5 (20.8) | 5 (14.7) | 0.370^f^ |
| Iron supplement | 4 (16.7) | 4 (11.8) | 0.436^f^ |

^f^ Fisher’s exact test; ^t^ unpaired t test; ^m^Mann-Whitney U test; MCHC: Mean corpuscular hemoglobin concentration, MCH: Mean corpuscular hemoglobin, MCV: Mean corpuscular volume; AST: aspartate aminotransferase; ALT: alanine aminotransferase.
