# Supplementary material for: The Peak Plasma Concentration (Cmax)/Minimum Inhibitory Concentration (MIC) of bedaquiline and levofloxacin with special attention to the sputum conversion in the treatment of multidrug-resistant tuberculosis in Indonesia
Source: PLoS One. 2025 Dec 31;20(12):e0336210. doi: 10.1371/journal.pone.0336210 (PMC12755803; doi:10.1371/journal.pone.0336210)
Supplement: S5 Table — (DOCX) [file pone.0336210.s005.docx]

**S5 Table. Demographic data and clinical characteristics of 30 MDR-TB patients at the start of treatment (baseline) who had Cmax/MIC data**

| **Characteristics** | **Conversion** | **Non Conversion** | **p value** |
| --- | --- | --- | --- |
|  | **(n = 26)** | **(n = 4)** |  |
| Age (years), median (min-max) | 42 (20–65) | 33 (26– 49) | 0.513^m^ |
| Sex, n (%) |  |  |  |
| Male | 20 (76.9) | 2 (50.0) | 0.284^f^ |
| Female | 6 (23.1) | 2 (50.0) |  |
| Previous TB treatment, n (%) | 15 (57.7) | 3 (75.0) | 0.469^f^ |
| Categorical BMI, n (%) |  |  |  |
| Normal weight (18.5 to 24.9 kg m^2^) | 12 (46.2) | 1 (25.0) | 0.409^f^ |
| Underweight (< 18.5 kg m^2^) | 14 (53.8) | 3 (75.0) |  |
| Lung lesion, n (%) |  |  |  |
| Moderate advanced | 3 (11.5) | 4 (100.0) | 0.087^m^ |
| Far advanced | 23 (88.5) | 0 |  |
| Laboratory results |  |  |  |
| Hemoglobin (g/dL), mean (SD) | 12 (2.1) | 11.5 (0.7) | 0.462^t^ |
| MCV MCV (fL), mean (SD) | 82 (6.9) | 77 (4.2) | 0.141^t^ |
| AST (IU/L), median (min-max) | 18.5 (10–62) | 16.5 (13–20) | 0.561^m^ |
| ALT (IU/L), median (min-max) | 16.0 (6–47) | 9.0 (7–20) | 0.087^m^ |
| Creatinine (mg/dL), mean (SD) | 0.67 (0.2) | 0.6 (0.2) | 0.534^t^ |
| Albumin (g/L), mean (SD) | 3.4 (0.6) | 3.2 (0.7) | 0.748^m^ |
| Other drug use, n (%) |  |  |  |
| Iron supplement | 24 (92.3) | 2 (50.0) | 0.075^f^ |

^f^ Fisher’s exact test; ^t^ unpaired t test; ^m^Mann-Whitney U test; AST: aspartate aminotransferase; ALT: alanine aminotransferase.
