# Supplementary material for: The Peak Plasma Concentration (Cmax)/Minimum Inhibitory Concentration (MIC) of bedaquiline and levofloxacin with special attention to the sputum conversion in the treatment of multidrug-resistant tuberculosis in Indonesia
Source: PLoS One. 2025 Dec 31;20(12):e0336210. doi: 10.1371/journal.pone.0336210 (PMC12755803; doi:10.1371/journal.pone.0336210)
Supplement: Attachment — Submitted filename: Response to Reviewers.-Plosone-Nina.docx [file pone.0336210.s007.docx]

| **No** | **Comments** | **Respons** | **Changes (lines) /Notes/Additional information** |
| --- | --- | --- | --- |
|  | **Journal Requirements:** |  |  |
| 1 | Please ensure that your manuscript meets PLOS ONE's style requirements, including those for file naming. The PLOS ONE style templates can be found at  File | We thank the editors for this mention. We have carefully reformatted the manuscript based on the PLOS ONE style template. |  |
| 2 | We note that the grant information you provided in the ‘Funding Information’ and ‘Financial Disclosure’ sections do not match.  When you resubmit, please ensure that you provide the correct grant numbers for the awards you received for your study in the ‘Funding Information’ section. | Apologies that it did not match. We carefully provide the correct information |  |
| 3 | Thank you for stating the following financial disclosure:  [Universitas Indonesia   <http://dx.doi.org/10.13039/501100006378>   NKB_292   Prof Purwanty Ascobat].  Please state what role the funders took in the study.  If the funders had no role, please state: "The funders had no role in study design, data collection and analysis, decision to publish, or preparation of the manuscript."  If this statement is not correct you must amend it as needed.  Please include this amended Role of Funder statement in your cover letter; we will change the online submission form on your behalf. | Prof Purwanty Ascobat is a supervisor and a team leader who applied for the Grant for this research.  Regarding the online submission form, we amended the statement.  We have no conflicts of interest to disclose.  The funders had no role in the study design, data collection, data analysis, manuscript preparation, or decision to publish. |  |
| 4 | Thank you for stating the following in your manuscript:  [This study was funded by the Directorate of Research and Development, Universitas Indonesia, under Hibah PUTI 2024 (Grant No NKB_292).]  We note that you have provided funding information that is not currently declared in your Funding Statement. However, funding information should not appear in the Acknowledgments section or other areas of your manuscript. We will only publish funding information present in the Funding Statement section of the online submission form.  Please remove any funding-related text from the manuscript and let us know how you would like to update your Funding Statement. Currently, your Funding Statement reads as follows:  [The author(s) received no specific funding for this work.]  Please include your amended statements within your cover letter; we will change the online submission form on your behalf. | We thank you for the correction. We changed the Funding Statement section of the online submission form. |  |
| 5 | In the online submission form, you indicated that [Data cannot be shared publicly due to restrictions. Data are available from the Institutional Data Access / Ethics Committee (contact via first author) for researchers who meet the criteria for accessing confidential data.  All PLOS journals now require all data underlying the findings described in their manuscript to be freely available to other researchers, either 1. In a public repository, 2. Within the manuscript itself, or 3. Uploaded as supplementary information.  This policy applies to all data except where public deposition would breach compliance with the protocol approved by your research ethics board. If your data cannot be made publicly available for ethical or legal reasons (e.g., public availability would compromise patient privacy), please explain your reasons on resubmission, and your exemption request will be escalated for approval. | We confirm that all relevant data are within the manuscript and its supporting information files. | Data Availability: All relevant data are within the manuscript and its supporting Information files. |
| 6 | When completing the data availability statement of the submission form, you indicated that you will make your data available on acceptance. We strongly recommend all authors decide on a data sharing plan before acceptance, as the process can be lengthy and hold up publication timelines. Please note that, though access restrictions are acceptable now, your entire data will need to be made freely accessible if your manuscript is accepted for publication. This policy applies to all data except where public deposition would breach compliance with the protocol approved by your research ethics board. If you are unable to adhere to our open data policy, please kindly revise your statement to explain your reasoning and we will seek the editor's input on an exemption. Please be assured that, once you have provided your new statement, the assessment of your exemption will not hold up the peer review process. | We confirm that all relevant data are within the manuscript and its supporting Information files.  Regarding the online submission form, we revised the statement. Thank you |  |
| 7 | Please amend the manuscript submission data (via Edit Submission) to include author Purwantyastuti Ascobat | Purwanty Ascobat is already included, but  we amended the manuscript submission data. |  |
| 8 | Please amend your authorship list in your manuscript file to include author Purwanty Ascobat. | Purwanty Ascobat is already included, but  We amended the manuscript submission data. |  |
|  |  |  |  |
|  |  |  |  |
|  | **Reviewers' comments:**  **Comments to the Author** |  |  |
| 1 | Is the manuscript technically sound, and do the data support the conclusions?  The manuscript must describe a technically sound piece of scientific research with data that supports the conclusions. Experiments must have been conducted rigorously, with appropriate controls, replication, and sample sizes. The conclusions must be drawn appropriately based on the data presented.  **Reviewer #1: Yes**  **Reviewer #2: Partly** | We thank the reviewer for the comments. We apologize if the data support may be partial. We have refined all sections to provide better context with data that support the conclusions. |  |
| 2 | Has the statistical analysis been performed appropriately and rigorously?  **Reviewer #1: Yes**  **Reviewer #2: No** | We thank the reviewer for the comments. We added the new figure and supplementary tables. Hopefully, these data will better balance transparency and supporting information | **Fig 3.** **Summary of the Outcome (conversion or non-conversion) based on Levofloxacin, Bedaquiline Cmax, and their minimum inhibitory concentrations (MICs) (A and B).**  **S1 File.** Sample size calculation output (screenshots and MedCalc parameters)  **S2 Table.** Demographic data and clinical characteristics of 58 MDR-TB patients at the start of treatment (baseline) by whether Bedaquiline or Levofloxacin Cmax is missing or not  **S3 Table.** Comparison of bedaquiline MIC and levofloxacin MIC with sputum conversion (n = 51)  **S4 Table.** Summary of assessing normality test using Shapiro-Wilk, skewness, and kurtosis for a characteristic of variables in various samples  **S5 Table.** Mean, confidence interval, median, variance of bedaquilin and levofloxacin cmax, cmax/mic ratio in MDR TB patients with sputum conversion and non-conversion groups  **S6 Table.** Additional baseline characteristics of 30 patients who had cmax/mic bedaquiline data |
| 3 | Have the authors made all data underlying the findings in their manuscript fully available?  The [PLOS Data policy](http://www.plosone.org/static/policies.action#sharing) requires authors to make all data underlying the findings described in their manuscript fully available without restriction, with rare exception (please refer to the Data Availability Statement in the manuscript PDF file). The data should be provided as part of the manuscript or its supporting information, or deposited to a public repository. For example, in addition to summary statistics, the data points behind means, medians and variance measures should be available. If there are restrictions on publicly sharing data—e.g. participant privacy or use of data from a third party—those must be specified.  **Reviewer #1: Yes**  **Reviewer #2: No** | We thank the reviewer for the suggestion. We added the new figure and the supplementary tables as supporting information. All relevant data are within the manuscript and its Supporting Information files. | Figure 3 and Supplementary tables (please see above ) |
| 4 | Is the manuscript presented in an intelligible fashion and written in standard English?  PLOS ONE does not copyedit accepted manuscripts, so the language in submitted articles must be clear, correct, and unambiguous. Any typographical or grammatical errors should be corrected at revision, so please note any specific errors here.  **Reviewer #1: Yes**  **Reviewer #2: No** | We thank the reviewer for the comments. We have carefully revised/reformatted the sentences or paragraphs (including any typographical or grammatical errors using Grammarly tools). | The revision was highlighted (yellow) in the manuscript |
| 5 | Review Comments to the Author  **Reviewer #1:** This study addresses an important public health issue in a high TB-burden setting by evaluating pharmacokinetic/pharmacodynamic (PK/PD) parameters, specifically the Cmax/MIC ratio of bedaquiline and levofloxacin, and their association with early sputum conversion in MDR-TB patients. The research is relevant and executed within clinical contexts reflective of Indonesian practice. However, several aspects require critical clarifications.  The number of evaluable patients for bedaquiline (n = 32) and levofloxacin (n = 48) Cmax measurements is relatively small and unbalanced between conversion and non-conversion groups, especially for bedaquiline (only 4 non-converters).  While the authors report a significant difference in bedaquiline Cmax/MIC between conversion and non-conversion groups, the small number of non-converters (n = 4) undermines confidence in the statistical validity of this finding. The assumption of normality for parametric tests (e.g., t-test) with such small group sizes is questionable.  The lack of significance in levofloxacin Cmax/MIC differences is presented without adequate discussion.  **Reviewer #2:** The authors looked at the extent to which the peak dosage at 1 month of 2 antibiotics given for multidrug-resistant tuberculosis (levofloxacin and bedaquiline) was associated with microbiological success (sputum negativity at 4 months). This is a relevant objective, and the data are interesting.  However, we are hampered by the absence of an essential piece of data: the minimum inhibitory concentrations for the 2 antibiotics according to the outcome (sputum conversion or not). MIC are described in general, but not per outcome.  The authors observe that the ratio between the maximal concentration and the MIC does indeed differ according to microbiological success, However, the variable most strongly associated with this success may be the MIC rather than the ratio Cmax/MIC.  The fact that the ratio peak/MIC is not different according to the microbiological sucess for levofloxacin is maybe due to the facts that 1) MIC for levofloxacin are for all but 2 cases under the breakpoint (1 mg/L), and 2) the concentration are not different according to outcome (11.4 vs 11.0 mg/L). Meanwhile, for bedaquiline, most of the strains are above the "clinical breakpoint" (see https://www.who.int/publications/i/item/WHO-CDS-TB-2018.5). Therefore, when the authors report the important difference in Cmaw according to the outcome, it would be far more clear if they also provide MIC. For example, a figure showing in the X axis the Cmax and in the Y axis the MIC, each case represented by a point, and with different point shape or color according to the outcome, may add clarity.  Moreover, the authors perform a peak concentration for levofloxacin as if it was a legitimate parameter for an antibiotic which they describe as dose-dependent, but, for fluoroquinolones, the parameter most often reported as relevant is the ratio between the air under the curve (AUC) and the MIC, and measuring AUC requires more than just one point.  In addition, the author report that 58 patients are analysed; meanwhile, they have data for Cmax and MIC for only 48 patients for levofloxacin, and 32 for bedaquiline. Why announce 58 in the abstract? In the same line, it is not appropriate to perform statistical tests with numbers so small (ex: non-conversion group: only 4 Cmax values for bedaquiline).  At last, multiple sentences should be reviewed, being structured in a strange way or lacking verb. | We divided the reviewer’s comments into several responses and each chaged (lines)   - We thank the reviewer for the assumption of normality with small group sizes. We assessed normality by Shapiro–Wilk and calculating skewness and kurtosis values and their standard errors. The absolute skewness and kurtosis ratios (value/SE) were < 2, This indicates the distribution of the sample of some variables was normal. - We added supplementary file sample size. The planned total enrollment was 57 patients, based on calculations that the largest number of Levofloxacin Cmax/MIC variable. This sample size yields 80% power to detect a relationship between levofloxacin Cmax/MIC and the proportion of patients who achieve sputum conversion. The ratio used was 1:2, estimating the difference in proportion between the two groups. These sample size assumptions were based on previously published studies that showed the difference of means between groups was small (for the Levofloxacin Cmax/MIC variable). Meanwhile, for the Bedaquiline Cmax/MIC variable, the difference of means between groups was large. Hence, it didn't require a large number of samples to detect a association of bedaquiline Cmax/MIC with sputum conversion. - We thank the reviewer for this insightful comment about unbalanced between conversion and non-conversion groups, especially bedaquiline Cmax/MIC variable.   We added it in discussion section, as well as supplementary table S5. Baseline characteristic of 30 patients.  This means that analyses conducted based on available data are less likely to produce biased results.  We also added the supplementary tables validity of our conclusions. We consider using parametrics test and non-parametric test, while the significant resuts was consistent. The variance between groups of bedaquiline Cmax/MIC was not homogeneous, the Welch's t-test was used (equal variances are not assumed) [1]   - We added more detail about the lack of significance in levofloxacin Cmax/MIC differences in disccusion suction   We divided the reviewer’s comments into several responses and each chaged (lines)   - We thank the reviewer for the comments of MIC. We have analyzed the comparison of bedaquiline MIC and levofloxacin MIC with Sputum Conversion.   We added a supplementary table: Comparison of Bedaquiline MIC and Levofloxacin MIC with Sputum Conversion (n = 51),   - We thank the reviewer for the suggestions. We have carefully reformat the manuscript and added the new figure. - We have stated information in the methods section that a total of 58 patients were observed for up to 4 months, while only 48 and 32 plasma samples were successfully analyzed for levofloxacin Cmax and Bedaquiline Cmax, respectively. We confirmed this in the sampling flow figure and the abstract. Meanwhile, we stated in the limitations study that   patients who had missing sample collection data (Cmax) for levofloxacin or bedaquiline.   - We stated that MICs for bedaquiline are also under the breakpoint (≤ 1 mg/L) in this study; it was not just the MIC for levofloxacin. The distribution of bedaquiline MICs was as follows: 21 (41.2%) at 0.25 mg/L, 18 (35.5%) at < 0.12 mg/L, and 12 (23.3%) at > 0.50 mg/L, including one MIC of 1 mg/L. That is why we mentioned that MICs for bedaquiline showed high pharmacodynamic variability. - We thank the reviewer for this insightful comment and consent on the imbalance between conversion and non-conversion groups. Our previous responses mentioned:   This unbalanced can be caused by consecutive patient enrollment and reflects the real-world distribution of cases during the study period, while the baseline characteristics of patients showed no significant difference between groups.  We added an S5 Table. Additional baseline characteristics of 30 patients who had Cmax/MIC bedaquiline data.  This means that analyses conducted based on available data are less likely to produce biased results.  We added the supplementary tables to validate our conclusions. We consider using parametric and non-parametric tests, while the significant results were consistent. The variance between groups of bedaquiline Cmax/MIC was not homogeneous; the Welch's t-test was used (equal variances are not assumed) [1]   - We thank the reviewer for the comments. We have carefully revised/reformatted the sentences or the structure of sentences to better convey the information | Lines 261 – 263:  S4 Table. Summary of Assessing normality test using Shapiro-Wilk, Skewness, Kurtosis for a characteristic of variables in various sizes of samples  Lines 261 – 263:  S5 Table. Mean, Confidence interval, Median, Variance of Bedaquilin and Levofloxacin Cmax, Cmax/MIC ratio in MDR TB Patients with Sputum Conversion and Non-Conversion groups.  Lines 126 – 127:  Additional sample size calculation output (screenshots and MedCalc parameters) is available in **S1 File**  Lines 274 – 277:  Additional baseline characteristics of 30 patients who had Cmax/MIC bedaquiline data are provided in **S6 Table**.  Lines 319 – 324:  However, there were 26 patients in the conversion groups and four patients in the non-conversion groups, resulting in an imbalance in the number of patients in different condition groups within a study. This imbalance can be caused by consecutive patient enrollment or reflects the real-world distribution of cases during the cohort study period. In general, the baseline characteristics of patients showed no significant difference between groups.  Lines 261 – 263:  Summary statistics for the mean, median, variance, and normality of the Cmax, Cmax/MIC data in the groups are available in **S4 Tables** and **S5 Tables.**  Lines 370 – 380: First, the high levofloxacin Cmax/MIC ratio alone may not significantly suggest the early sputum conversion, as the variability in the distribution of levofloxacin MIC may be lower than that of bedaquiline MIC. Second, the Cmax of levofloxacin was not different according to the clinical outcome. Third, the limitation in the number of study samples for the levofloxacin Cmax/MIC variable may not capture the clinical variability; therefore, a larger number of patients is needed to validate these findings. Fourth, the area under the curve (AUC) of plasma concentration and the MIC might be a more relevant parameter to further ensure the clinical outcome. However, AUC plasma concentration measurement requires more than one point sampling, which cannot be performed in this study due to technical limitations [2,3]. Fifth, there was a delay in sputum conversion caused by inadequate exposure of other anti-TB drugs, such as bedaquiline Cmax in the regimen  Lines 241 – 243:  Results section:  The MIC values for bedaquiline and levofloxacin, based on clinical outcome (sputum conversion or non-conversion), did not show significant differences (see S3 Table).  Lines 329 – 332  Discussion section:  Meanwhile, MIC alone could not be compared with the outcome (sputum conversion), which did not provide more information than the pharmacokinetic/pharmacodynamic (Cmax/MIC) approach, because it encompasses the interaction between the host, pathogen, and medication.  Lines 274 – 280  Results section  **Fig. 3** shows a summary of outcome (conversion or non-conversion) based on levofloxacin, bedaquiline Cmax, and their MIC.  Lines 208:  **Fig. 1**. This is Fig. 1. Patient recruitment and follow-up flowchart  Lines 200 – 205  Caption of Fig.1. A total of 74 patients who met the inclusion criteria and did not meet the exclusion criteria were enrolled from new patients treated with MDR-TB regimens in December 2023 and September 2024 at three hospitals. Fifty-eight patients were successfully followed up to four months of treatment and completed analysis. Forty-eight plasma samples were successfully analyzed for levofloxacin Cmax, and 32 plasma samples for bedaquiline Cmax.  Lines 218 – 220  Additional baseline characteristics of the group with missing bedaquiline or levofloxacin Cmax data and the group without missing Cmax data are provided in **S2 Table**.  Lines 393 – 395  Discussion section  This study has several limitations. First, this study had some missing Cmax data for bedaquiline or levofloxacin due to technical factors; however, there were no significant differences between patients with missing data and those without missing data.  Lines 238 – 241  In this study, MIC bedaquiline 0.25 mg/L was 21 (41.2%) Mtb isolates, MIC ≤ 0.12 mg/L was 18 (35.5%) isolates, and MIC ≥ 0.50 mg/L was 12 (23.3%) isolates. Meanwhile, the MICs of levofloxacin against Mtb of 0,25 mg/L were 35 (68.6%) isolates (**Fig 2**).  Lines 325 – 327  MIC distribution of bedaquiline for Mtb was presented in this study, which showed high pharmacodynamic variability  Highlighted yellow |
| 6 | PLOS authors have the option to publish the peer review history of their article ([what does this mean?](https://journals.plos.org/plosone/s/editorial-and-peer-review-process#loc-peer-review-history)). If published, this will include your full peer review and any attached files.  If you choose “no”, your identity will remain anonymous but your review may still be made public.  **Do you want your identity to be public for this peer review?** For information about this choice, including consent withdrawal, please see our [Privacy Policy](https://www.plos.org/privacy-policy).  **Reviewer #1: No**  **Reviewer #2: No** | We appreciate the reviewer's answer |  |
|  |  |  |  |
|  |  |  |  |

1.	West RM. Best practice in statistics: Use the Welch t-test when testing the difference between two groups. Ann Clin Biochem. 2021;58(4):267–9.

2
