# Supplementary material for: The Peak Plasma Concentration (Cmax)/Minimum Inhibitory Concentration (MIC) of bedaquiline and levofloxacin with special attention to the sputum conversion in the treatment of multidrug-resistant tuberculosis in Indonesia
Source: PLoS One. 2025 Dec 31;20(12):e0336210. doi: 10.1371/journal.pone.0336210 (PMC12755803; doi:10.1371/journal.pone.0336210)
Supplement: Attachment — Submitted filename: Responses to reviewer rounds2 Plosone.docx [file pone.0336210.s008.docx]

Response to reviewers (rounds 2)

| No | Reviewers' comments: | Responses/Additional information | Changes (lines) /Notes |
| --- | --- | --- | --- |
| 1 | If the authors have adequately addressed your comments raised in a previous round of review and you feel that this manuscript is now acceptable for publication, you may indicate that here to bypass the “Comments to the Author” section, enter your conflict of interest statement in the “Confidential to Editor” section, and submit your "Accept" recommendation.  Reviewer #1: All comments have been addressed  Reviewer #2: (No Response) | We thank the reviewer for the continued feedback, which we believe has helped us improve the clarity and accuracy of our manuscript.  We apologize if our previous revision did not fully address all the points raised. In the current version, we have revised the manuscript point-by-point based on the responses. We hope this revision addresses the reviewers' concerns. |  |
| 2 | Is the manuscript technically sound, and do the data support the conclusions?  The manuscript must describe a technically sound piece of scientific research with data that supports the conclusions. Experiments must have been conducted rigorously, with appropriate controls, replication, and sample sizes. The conclusions must be drawn appropriately based on the data presented.  Reviewer #1: Yes  Reviewer #2: Yes | We thank the reviewer for the comments |  |
| 3 | Has the statistical analysis been performed appropriately and rigorously?  Reviewer #1: Yes  Reviewer #2: No | We thank the reviewers for the continued feedback and suggestions to improve statistical rigor and believe the revised manuscript now addresses these issues. |  |
| 4 | Have the authors made all data underlying the findings in their manuscript fully available?  The PLOS Data policy requires authors to make all data underlying the findings described in their manuscript fully available without restriction, with rare exception (please refer to the Data Availability Statement in the manuscript PDF file). The data should be provided as part of the manuscript or its supporting information, or deposited to a public repository. For example, in addition to summary statistics, the data points behind means, medians and variance measures should be available. If there are restrictions on publicly sharing data—e.g. participant privacy or use of data from a third party—those must be specified.  Reviewer #1: Yes  Reviewer #2: Yes | We thank the reviewer for the comments |  |
| 5 | Is the manuscript presented in an intelligible fashion and written in standard English?  PLOS ONE does not copyedit accepted manuscripts, so the language in submitted articles must be clear, correct, and unambiguous. Any typographical or grammatical errors should be corrected at revision, so please note any specific errors here.  Reviewer #1: Yes  Reviewer #2: No | We have carefully revised/reformatted the sentences or paragraphs | The revision was highlighted (yellow) in the manuscript |
| 6 | Review Comments to the Author |  |  |
|  | Reviewer #1: The number of samples were increased in supplement section in all conditions. Also, 3. the lack of significance in levofloxacin Cmax/MIC differences is now presented in discussion. Thus, all concerns are well addressed in the revised version. | We thank the reviewer for the comments and for carefully reviewing our previously revised manuscript. |  |
|  | Reviewer #2: The authors have addressed most of the comments from the reviewers.  This is important, as the data reported deserve publication.  There is still some issues with some points:  for the abstract:  - "58 patients completed the analysis at four months of treatment.": again, no. Your article title is on antibiotic concentration, so you cannot say that 58 patients completed the study if 10 of them did not. The sentence that corrects this figure just after is a point, but you should straightforward say 48, not 58.  - "There was a statistically significant difference between the Cmax/MIC ratio of bedaquiline in the sputum conversion and non-conversion groups (9.10 vs. 1.65; p < 0.001).": again, you cannot do a statistical analysis with 3 or 4 patients in one of the group. | We thank the reviewer for the suggestions. We have carefully reformat the sentence.  We have carefully reformat the sentence in the abstract section and added information in the discussion section.  Our study was designed with prior sample size calculations for each Cmax/MIC variable of interest based on an existing study. So, we exceeded the target sample size for the variable Cmax/MIC bedaquiline that could be evaluated. This ensured adequate power for each primary analysis. However, the small number of patients in the non-conversion group (n=4) reflects real-world distribution and requires careful interpretation. | Lines 58–61  Among the 74 patients enrolled, 16 dropped out during the four-month follow-up period. Blood samples were successfully obtained 1–2 hours after drug administration from 48 patients and 4–6 hours after drug administration from 32 patients, which were used for pharmacokinetic analysis.  Lines 62–63  The mean Cmax/MIC ratio of bedaquiline was higher in the sputum conversion group compared with the non-conversion group (9.10 vs. 1.65, respectively).  Lines 330–335  However, 26 patients who had Cmax/MIC of bedaquiline in the conversion groups and four patients in the non-conversion groups (26 vs. 4) resulted in an imbalance in the number of patients in different condition groups within a study. This imbalance can be caused by consecutive patient enrollment or reflects the real-world distribution of cases during the cohort study period. In general, the baseline characteristics of patients showed no significant difference between groups [27].  Lines 419–422  However, the possibility of implementation of this plasma concentration monitoring in clinical practice should be carefully considered and supported by further research in various settings. |
|  | for the text:  - "The MIC values for bedaquiline and levofloxacin, based on clinical outcome (sputum conversion or non-conversion), did not show significant differences (see S3 Table).": I is a good thing that the authors added this data. However, this is a major data, it should not be in a supplementary table, but as a part of the main table describing the particularities of the patients included.  - "In this study, MIC bedaquiline 0.25 mg/L was 21 (41.2%) Mtb isolates,": this fragment sentence makes no sense, a word or a mathematical sign is lacking.  - "Meanwhile, MIC alone could not be compared with the clinical outcome (sputum conversion)": this sentence is not clear. What would one want to compare a MIC with non-MIC data? Maybe the authors want to say " Groups made according to MIC alone could not be compared with the clinical outcome (sputum conversion)"; but if it is what they had in mind, I do not understand why - it is basic practice to see whether people infected with a bacterial strain with high MIC have or not an outcome different from people infected wiht a bacterial strain with lower MIC.  - "MIC distribution of bedaquiline for Mtb was presented in this study, which showed high pharmacodynamic variability.": it is a bit confusing to say so, as pharmacodynamic also result from PK. Moreover, there is not "pharmacodynamic variability"; conversely, pharmacodynamic analyses lead to variable conclusions according to PK and to MIC  - "According to the MIC of levofloxacin, most of the MICs of levofloxacin against Mtb in this study were 0.25 mg/L (68.6%)": the sentence makes no sense; maybe the authors want to say "Regarding the MIC of levofloxacin, most (68.6%) of the Mtb strains in this study were under the value of 0.25 mg/L" (or "were equal to 0.25 mg/L"). | We have added this information in the results section  We have carefully reformat this sentence to convey the idea more clearly.  We thank the reviewer for the corrections. We have carefully reformat this sentence to convey the idea more clearly, and based on your suggestions  We thank the reviewer for the corrections | Lines 249– 252  Table 2.  Lines 237–240  In this study, the MIC of bedaquiline showed that 41.2% of Mtb strains were equal to 0.25 mg/L; 35.5% of strains were under 0.12 mg/L, and 23.3% of strains were ≥ 0.50 mg/L. Meanwhile, regarding the MIC of levofloxacin, most (68.6%) of the Mtb strains in this study were equal to 0.25 mg/L (Fig 2).  Lines 336–343  The magnitude of the MIC value and the pharmacokinetic/pharmacodynamic parameter of bedaquiline will impact the clinical outcomes [28]. In this study, we evaluated whether patients infected with Mtb strains that had higher MIC values might have different outcomes compared to patients infected with Mtb strains that had lower MIC values. Meanwhile, there was no significant difference between the group with MIC < 0.25 mg/L and the group with MIC ≥ 0.25 mg/L in comparison to sputum conversion. The existing MIC grouping may not provide more information than the pharmacokinetic/pharmacodynamic (Cmax/MIC) approach, because this approach encompasses interactions between the host, pathogen, and drug  Lines 239–240  Meanwhile, regarding the MIC of levofloxacin, most (68.6%) of the Mtb strains in this study were equal to 0.25 mg/L |
|  | for the new figure 3: remove the lines of "correlation": the idea was not to see a correlation (this would be completely nonsense in this case), but to better visualise the data. (Moreover, it is not possible to draw a correlation line with 3 dots...) | We thank the reviewer for the corrections. We assess a new figure | Lines 287–289  Fig 3 |
| 7 | PLOS authors have the option to publish the peer review history of their article (what does this mean?). If published, this will include your full peer review and any attached files.  If you choose “no”, your identity will remain anonymous but your review may still be made public.  Do you want your identity to be public for this peer review? For information about this choice, including consent withdrawal, please see our Privacy Policy.  Reviewer #1: No  Reviewer #2: No | We appreciate the reviewer's comment |  |
